# Parathyroid Carcinoma: A Report of Two Cases

**DOI:** 10.7759/cureus.115709

**Published:** 2026-09-03

**Authors:** Cyrilkumaar Vijayakumar, Archit Dhanasekar, Ganapathy Dhanasekar

**Affiliations:** 1 Otolaryngology, Scunthorpe General Hospital, Northern Lincolnshire and Goole Hospitals NHS Trust, Scunthorpe, GBR; 2 Otolaryngology, University of Leicester, Leicester, GBR; 3 Otolaryngology - Head and Neck Surgery, Scunthorpe General Hospital, Northern Lincolnshire and Goole Hospitals NHS Trust, Scunthorpe, GBR

**Keywords:** case report, hoarseness, hypercalcaemia, parathyroid carcinoma, parathyroidectomy, primary hyperparathyroidism, sestamibi scan

## Abstract

Parathyroid carcinoma is a rare cause of hypercalcaemia, which can be potentially difficult to diagnose and treat. Since its clinical, biochemical, and radiological features overlap with those of parathyroid adenoma, diagnosis is often made only after histological review of the resected tissue. We report two cases managed at a United Kingdom district general hospital. The first case was of a 51-year-old man referred with persistent hoarseness and an incidental parathyroid lesion on imaging, in whom progressive hypercalcaemia led to excision and a histological diagnosis of parathyroid carcinoma. The second case was of a 58-year-old woman with recurrent hypercalcaemia after initial parathyroidectomy for presumed adenoma; a second resection and specialist histopathological review established the diagnosis of parathyroid carcinoma. Both patients achieved biochemical remission after definitive surgery, with no evidence of recurrence on subsequent long-term follow-ups. These cases illustrate the diagnostic difficulty posed by parathyroid carcinoma and underscore the importance of considering it in patients with marked or recurrent hypercalcaemia, particularly when associated with very high parathyroid hormone levels or local symptoms such as voice change.

## Introduction

Primary hyperparathyroidism is a common endocrine disorder with an estimated incidence of one to four cases per 1,000 in the general population [1,2]. Most cases arise from benign parathyroid adenoma, while parathyroid carcinoma is responsible for less than 1% of cases [2]. Fewer than 400 cases had been reported worldwide as of 2008, reflecting both the rarity of the condition and the diagnostic challenge it presents [3].

Parathyroid carcinoma typically presents with severe hypercalcaemia and markedly elevated parathyroid hormone (PTH) and may additionally cause a palpable neck mass, bony disease, renal stones, or recurrent laryngeal nerve involvement manifesting as hoarseness [3]. Preoperative distinction from adenoma is often impossible, and definitive diagnosis depends on histological evidence. The clinical importance of the distinction lies in the natural history of the disease: approximately 30% of patients experience local recurrence, and a further 30% develop metastases to regional lymph nodes, lungs, liver, or bone [2].

We report two cases of parathyroid carcinoma diagnosed and managed at a district general hospital over a five-year period. Both illustrate the diagnostic difficulty of this malignancy and the role of definitive surgical excision in achieving biochemical and clinical remission.

## Case presentation

Case 1

A 51-year-old man was referred by his general practitioner to the Ear, Nose, and Throat (ENT) department with persistent, unexplained hoarseness of voice lasting three weeks, which had developed following a flu-like illness. He worked as a paramedic, was a non-smoker, and reported no weight loss, haemoptysis, dysphagia, or neck lumps. General examination was unremarkable besides a croaky voice, with no palpable lymphadenopathy or cervical swelling.

Flexible laryngoscopy demonstrated a left vocal cord palsy. Computed tomography (CT) of the neck and chest was unremarkable except for a left-sided nodule posterior to the thyroid lobe, suggestive of a parathyroid adenoma. The patient was referred to speech and language therapy for his hoarseness. Following this, an ultrasound of the neck identified a well-defined hypoechoic lesion, measuring 15 × 13 × 12 mm, separate from the thyroid gland. A subsequent sestamibi subtraction scan confirmed a focus of tracer uptake in the left upper parathyroid position, consistent with a parathyroid lesion (Figures 1, 2).

**Figure 1:**
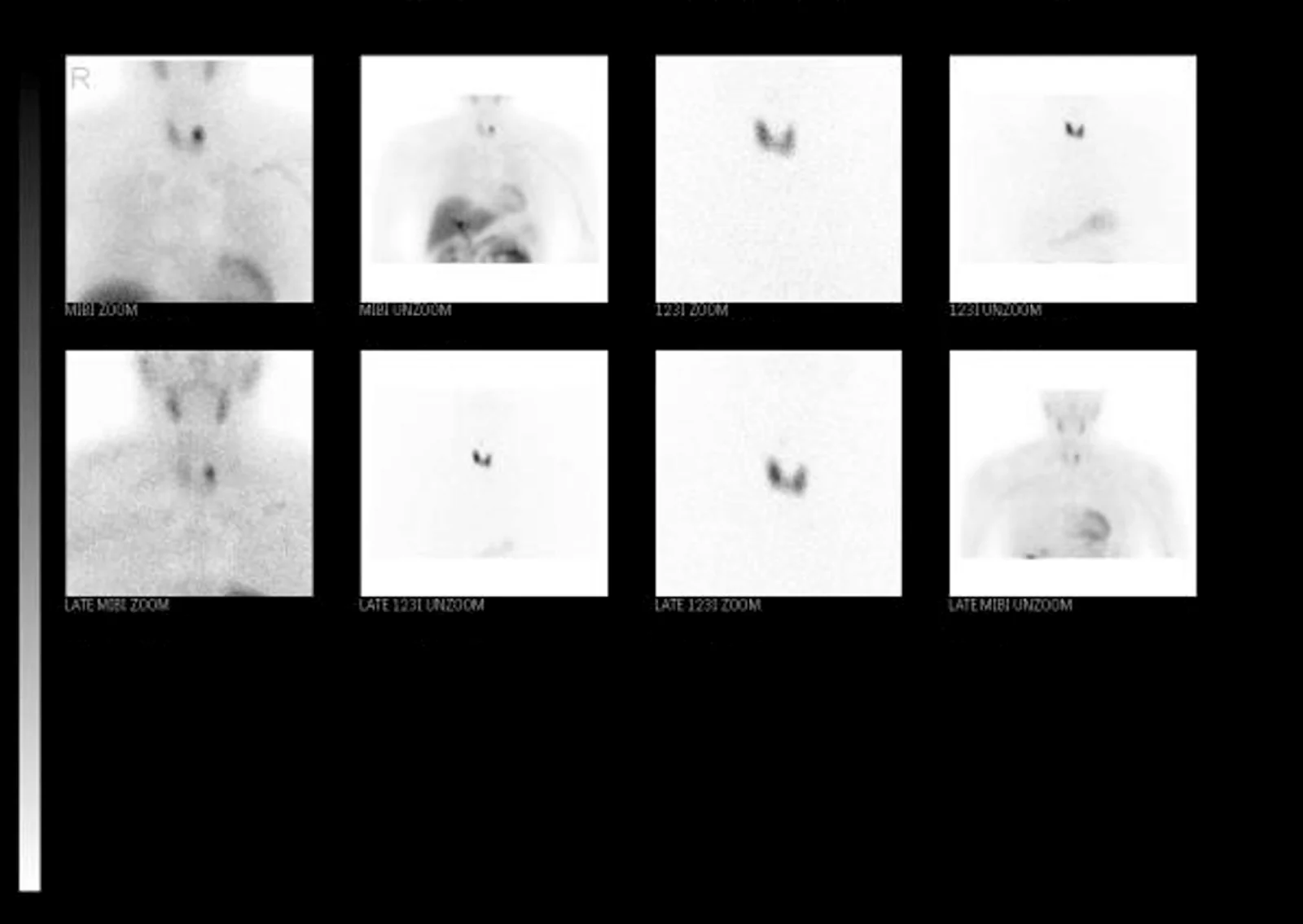
Sestamibi subtraction scan (case 1): findings consistent with a parathyroid lesion in the left upper position

**Figure 2:**
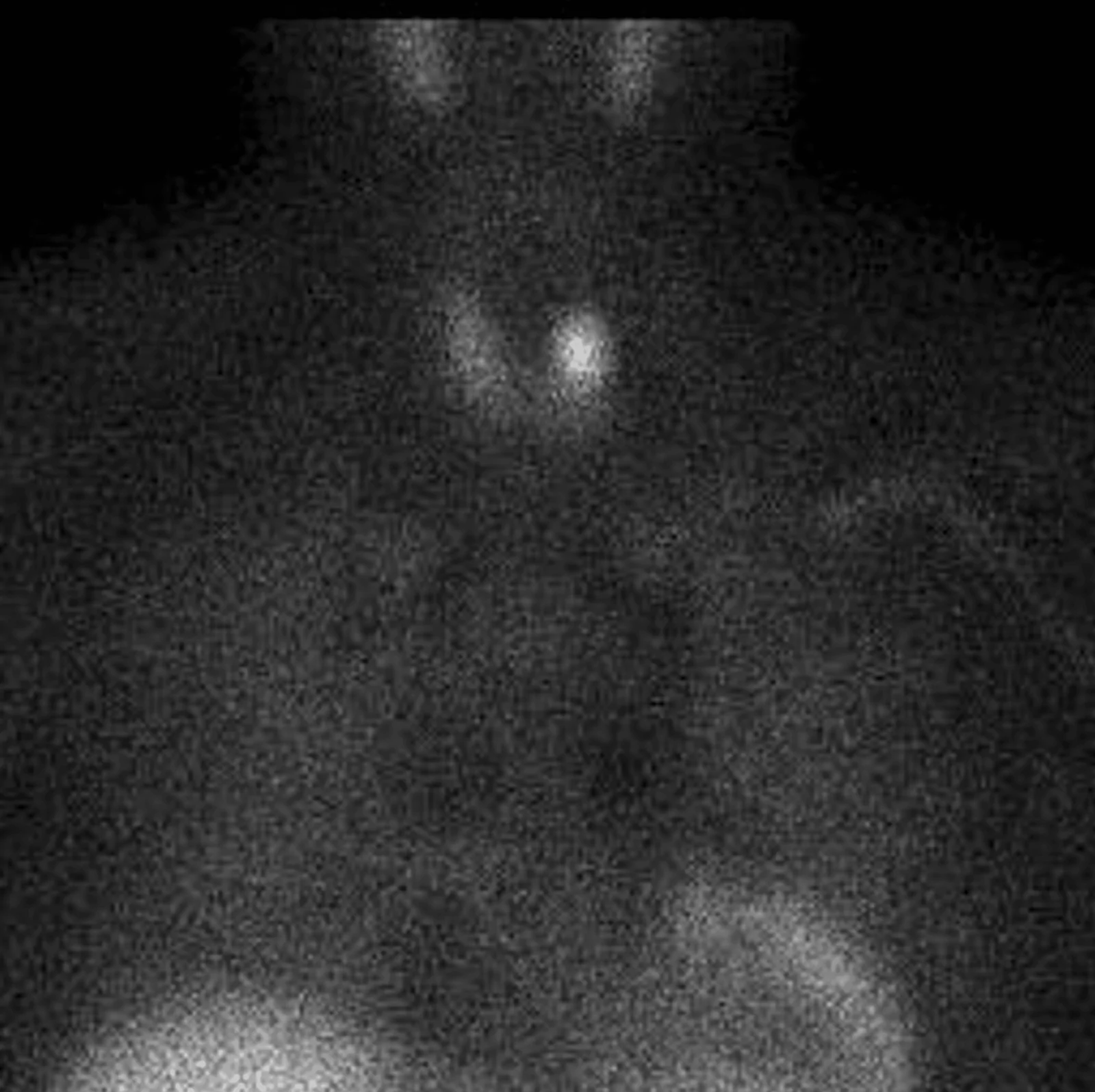
Sestamibi parathyroid subtraction image (case 1): close-up view highlighting the focal tracer retention in the left upper parathyroid position

During endocrinology follow-up, serum adjusted calcium and PTH continued to rise. Cinacalcet 30 mg twice daily was commenced approximately two years after presentation; cinacalcet acts as a calcimimetic, reducing PTH secretion and consequently serum calcium [4]. Screening for multiple endocrine neoplasia type 1 (MEN1) was negative.

Approximately three years after presentation, the patient underwent exploration and excision of the presumed left parathyroid adenoma. Postoperative recovery was uncomplicated, and biochemical parameters normalised (Table 1). The chronological sequence of investigations leading to diagnosis is summarised in Table 2.

**Table 1:** Serial biochemical results for case 1 Ca, calcium; PTH, parathyroid hormone; TSH, thyroid-stimulating hormone; T3, triiodothyronine; T4, thyroxine; N/A, not available

Parameter	Presentation	Month 13	Month 20	Month 25	Month 28	Month 36 (post-op day 0)	Month 36 (post-op day 1)	Reference range
Adjusted Ca (mmol/L)	2.83	2.48	2.9	2.79	2.73	2.72	2.54	2.20–2.60
Phosphate (mmol/L)	0.88	1.03	0.79	0.76	0.97	0.47	1.18	0.80–1.50
PTH (pmol/L)	9.9	6.4	12.5	14.4	N/A	1.1	1.9	1.6–6.9
TSH (mU/L)	2.1	N/A	2.2	2.1	N/A	N/A	N/A	0.27–4.5
T3 (pmol/L)	N/A	N/A	4.14	N/A	N/A	N/A	N/A	3.1–6.8
T4 (pmol/L)	N/A	N/A	13	N/A	N/A	N/A	N/A	11–23

**Table 2:** Chronological investigation timeline for case 1 MEN1, multiple endocrine neoplasia type 1; CT, computed tomography

Date	Investigation	Findings
Presentation	CT of the neck and chest	Suggestive of parathyroid adenoma. Two small (2 mm) nodules in the lower lobes of both lungs, of uncertain significance.
Month 1	Ultrasound neck	Well-defined hypoechoic lesion at the posterior aspect of the left thyroid lobe, 15 × 13 × 12 mm, separate from the thyroid gland.
Month 4	Parathyroid subtraction (sestamibi) scan	Imaging consistent with parathyroid adenoma in the left upper position (Figures 1, 2).
Month 26	MEN1 genetic screening	Negative.
Month 37	Histology of excised parathyroid gland	Highly suggestive of parathyroid carcinoma.
Month 38 (post-operative)	CT of the neck and chest (post-operative)	No evidence of local recurrence. No lymphadenopathy. Chest clear.

Histological examination of the resected specimen was highly suggestive of parathyroid carcinoma (Figure 3). The case was discussed at the regional thyroid multidisciplinary team (MDT) meeting, and re-exploration was recommended to ensure complete excision. This was performed at a tertiary centre. Postoperative CT of the neck and chest demonstrated no residual tumour and no lymphadenopathy. The patient remains under regular clinical follow-up.

**Figure 3:**
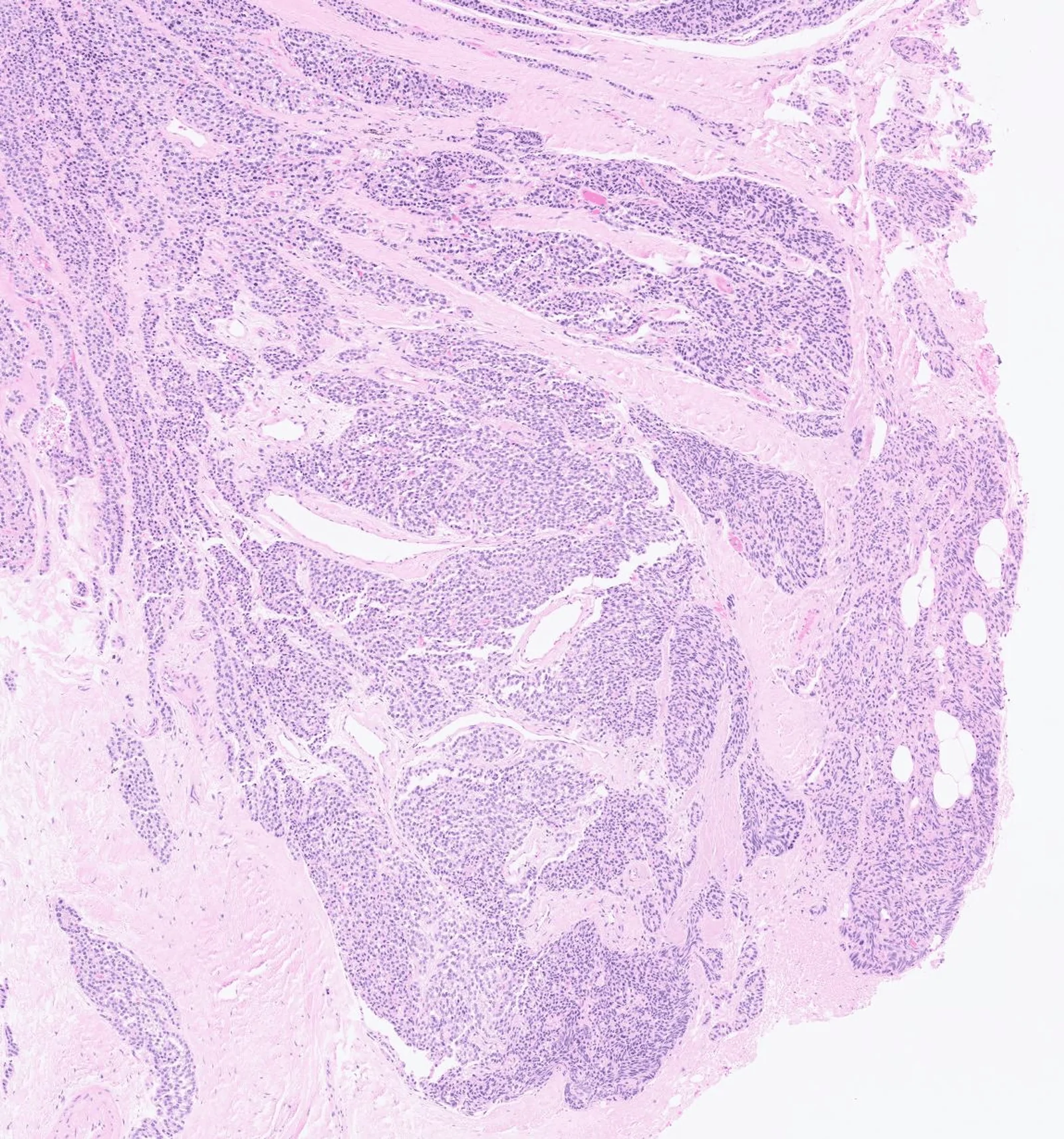
Haematoxylin and eosin-stained section of the resected parathyroid specimen (Case 1): tumour infiltrating the capsule and adjacent adipose tissue (original magnification ×5)

Case 2

A 58-year-old woman was referred to the ENT clinic with hyperparathyroidism and hypercalcaemia. Neck ultrasound revealed a 20 × 18 mm hypoechoic lesion in the inferior aspect of the left thyroid lobe. Five months after presentation, she underwent surgical exploration and excision of the suspected left inferior parathyroid adenoma. Her symptoms improved, and histology showed benign hyperplasia with no evidence of malignancy; however, serum calcium and PTH levels remained elevated. MEN syndrome was considered as a possible differential.

Approximately 11 months after presentation, symptomatic hypercalcaemia recurred. Sestamibi scanning the following month demonstrated reactivation of the left lower parathyroid gland (Figures 4, 5). A repeat ultrasound and sestamibi scan approximately 14 months after presentation were again consistent with a left parathyroid adenoma. Approximately two years after presentation, the patient underwent re-exploration of the left lower parathyroid gland. The tumour was located in the tracheo-oesophageal groove and extended deeply, making dissection technically challenging, but it was completely excised.

**Figure 4:**
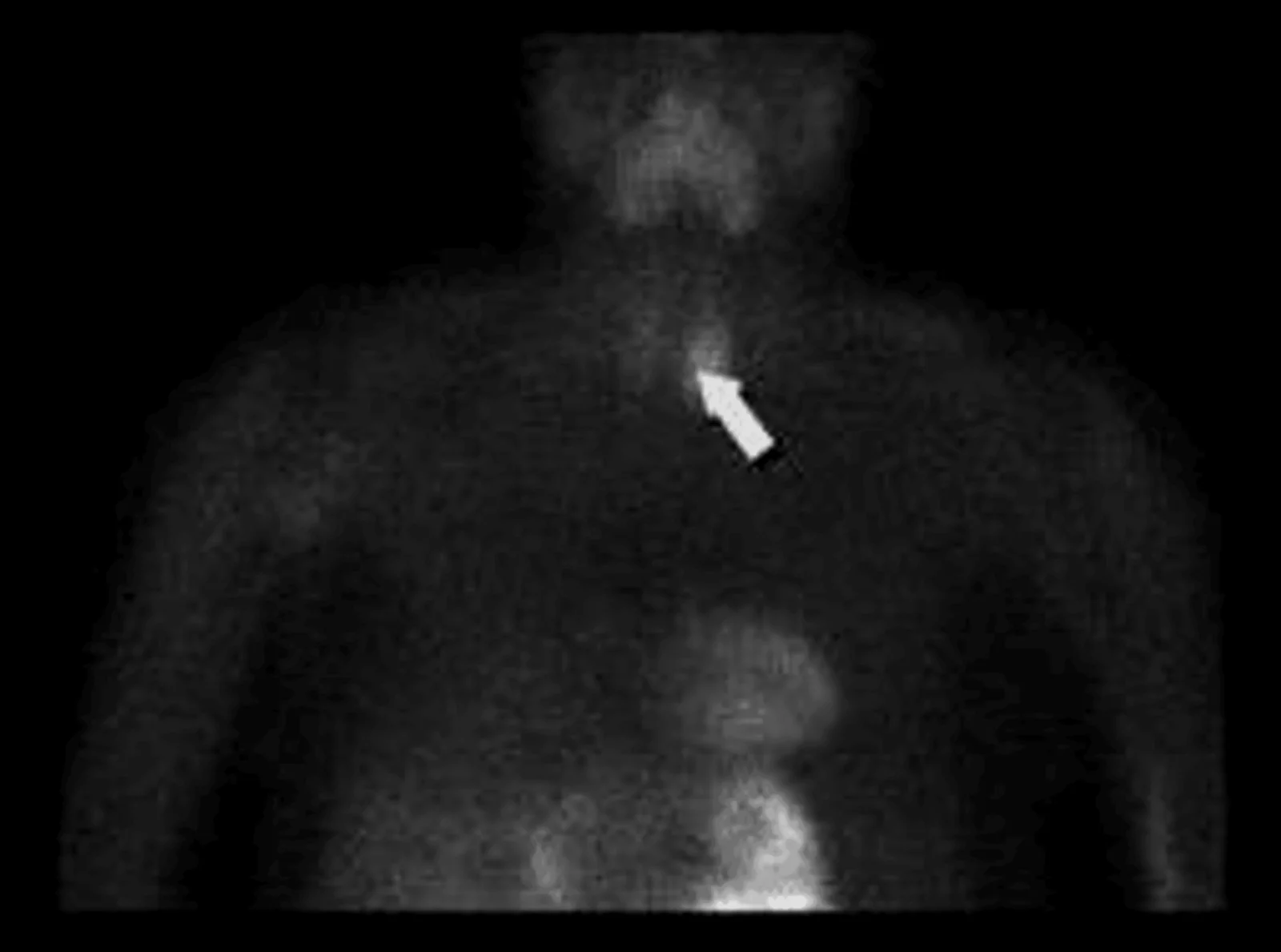
Sestamibi subtraction scan (case 2): close-up image showing tracer uptake in the left lower parathyroid gland (arrow), indicating reactivation of the left lower parathyroid gland

**Figure 5:**
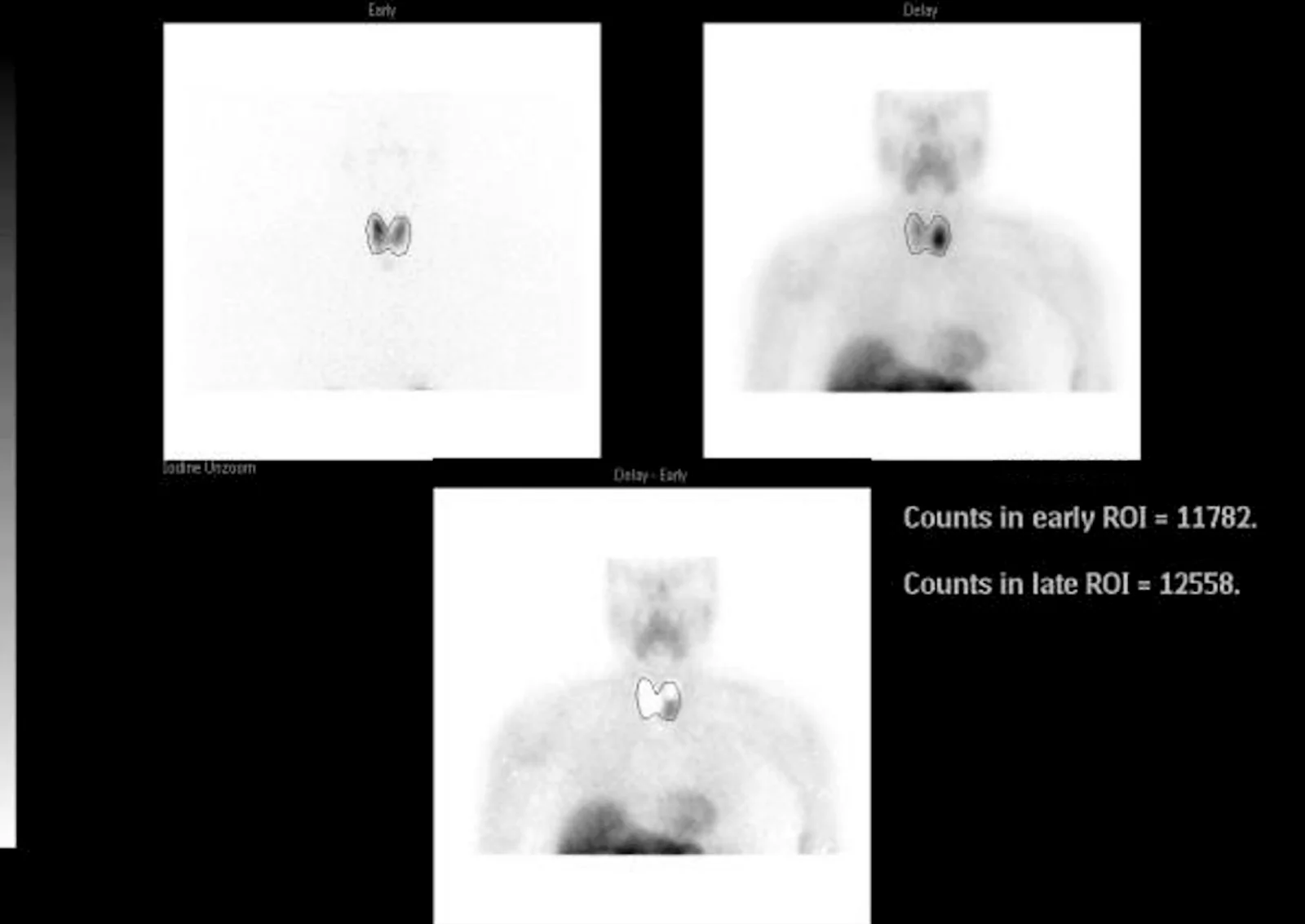
Sestamibi subtraction scan (case 2): tracer retention in the left lower parathyroid position Early and delayed acquisitions with ROI analysis (early counts = 11,782; late counts = 12,558). ROI, region of interest

Histopathology of the resected specimen showed proliferative parathyroid tissue, and a specialist opinion was sought from a tertiary histopathology centre; this review confirmed parathyroid carcinoma (Figure 6). The case was discussed at the thyroid MDT.

**Figure 6:**
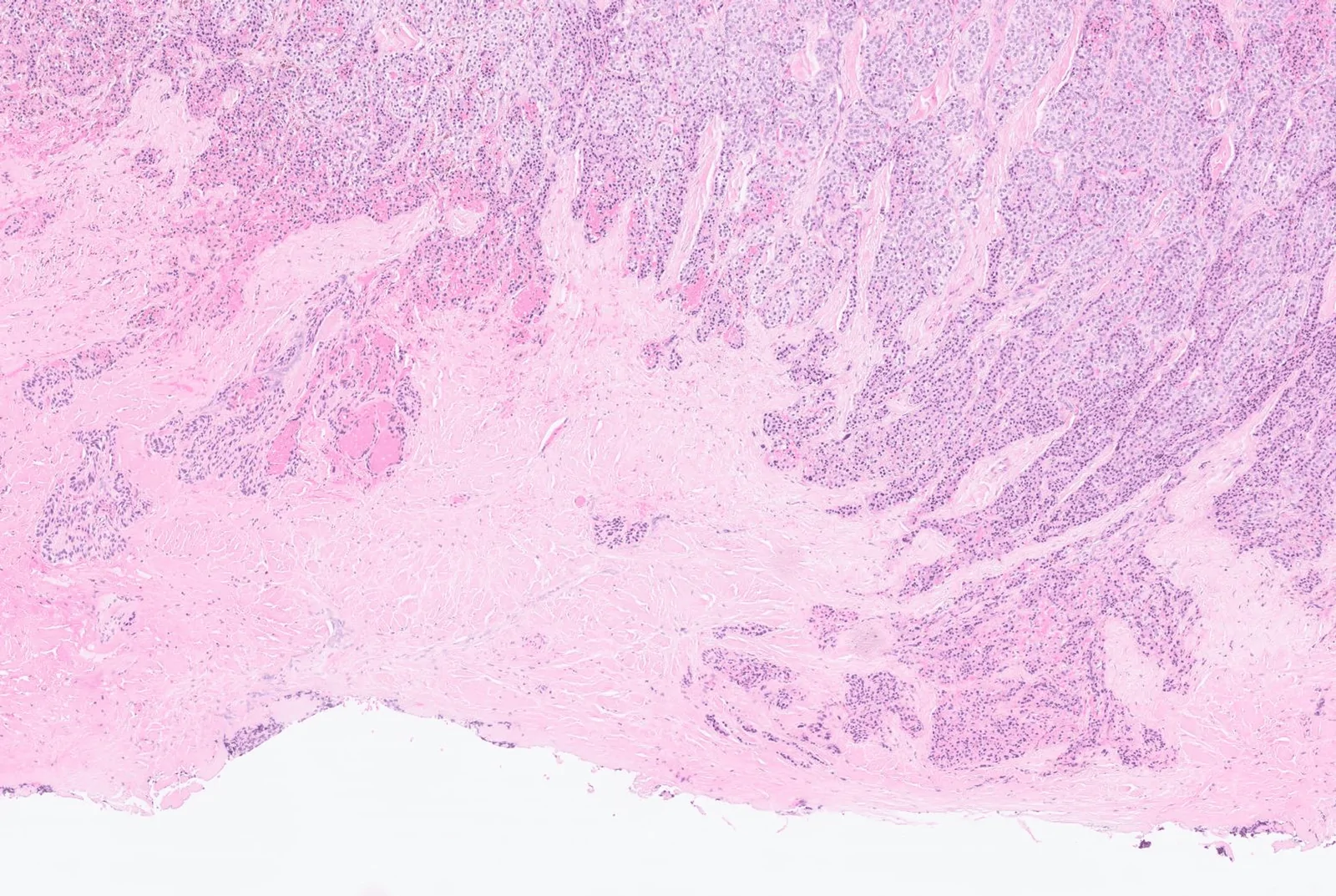
Haematoxylin and eosin-stained section of the resected parathyroid specimen (case 2): tumour deeply infiltrating the capsule (original magnification ×5)

Follow-up blood tests approximately two and a half years after presentation demonstrated normalisation of calcium and PTH (Table 3). CT of the thorax approximately three years after presentation showed no features of concern, and subsequent outpatient reviews have shown sustained biochemical remission with no clinical or radiological evidence of recurrence. The chronological sequence of investigations for this case is summarised in Table 4.

**Table 3:** Serial biochemical results of case 2 Ca, calcium; PTH, parathyroid hormone; TSH, thyroid-stimulating hormone; T3, triiodothyronine; T4, thyroxine; N/A, not available

Parameter	Month 4	Month 7	Month 11	Month 14	Month 23 (pre-op)	Month 23 (post-op)	Month 30	Reference range
Adjusted Ca (mmol/L)	3.31	2.57	2.74	2.87	2.47	2.52	2.52	2.20–2.60
Phosphate (mmol/L)	0.69	0.79	0.75	0.69	0.70	0.96	0.77	0.80–1.50
PTH (pmol/L)	N/A	39.0	21.0	18.5	66.9	9.8	6.5	1.6–6.9
TSH (mU/L)	6.8	0.45	0.33	N/A	N/A	N/A	N/A	0.27–4.5
T3 (pmol/L)	N/A	3.09	N/A	N/A	N/A	N/A	N/A	3.1–6.8
T4 (pmol/L)	14.5	12.7	N/A	N/A	N/A	N/A	N/A	11–23

**Table 4:** Chronological investigation timeline of case 2 PTH, parathyroid hormone; CT, computed tomography

Date	Investigation	Findings
Presentation	Ultrasound neck	Hypoechoic lesion, 20 × 18 mm, in the inferior aspect of the left thyroid lobe.
Month 5	Histology of initial surgical exploration	Benign hyperplasia. No evidence of malignancy.
Month 12	Sestamibi scan	Reactivation of the left lower parathyroid gland.
Month 14	Repeat ultrasound and sestamibi scan	Suggestive of left parathyroid adenoma.
Month 23	Histology of excision of left lower parathyroid gland	Proliferative parathyroid tissue.
Month 26	Newcastle supplementary histology report	Parathyroid carcinoma.
Month 29	Follow-up blood tests	Normalisation of PTH and calcium (Table 3). Subsequent follow-up remained normal.
Month 33	CT of the thorax	No features of clinical concern. Further follow-up showed no evidence of recurrence.

## Discussion

The two cases presented here highlight the diagnostic challenge posed by parathyroid carcinoma. Neither patient presented with features specific to malignancy. In case 1, an incidental parathyroid lesion was identified during investigation of an unrelated episode of hoarseness, while case 2 presented with biochemical features indistinguishable from a benign adenoma. In both cases, the diagnosis of carcinoma was made only after histological review of the resected specimen, and in case 2, it was made only after specialist pathology opinion following initial surgery.

Parathyroid carcinoma classically produces more pronounced biochemical derangement than adenoma. Serum calcium above 14 mg/dL (approximately 3.5 mmol/L) and PTH levels more than twice the upper limit of normal are considered suggestive [5]. Patients may also develop symptoms related to hypercalcaemia, including pathological fractures, bone and joint pain, fatigue, polyuria, polydipsia, nephrolithiasis, muscular weakness, nausea, anorexia, and weight loss [5]. Case 2 demonstrated a peak adjusted calcium level of 3.31 mmol/L and a PTH level more than nine times the upper reference limit prior to definitive surgery, a combination that should raise suspicion for malignancy. Local symptoms such as voice change, as seen in case 1, may reflect recurrent laryngeal nerve involvement and should be treated as a red flag.

Imaging has a supportive rather than definitive role. Neck ultrasound is typically the first-line localisation modality, complemented by technetium-99m sestamibi scintigraphy and, where indicated, CT imaging to evaluate local invasion or distant spread. However, no imaging modality can reliably distinguish carcinoma from adenoma preoperatively.

Histology remains the only definitive means of diagnosis. Features suggestive of malignancy include vascular invasion, capsular invasion, dense fibrous bands, and increased mitotic activity [1,6]. Even so, microscopic diagnosis is difficult; mitotic activity may be observed in adenomas, and some tumours with such features are classified as atypical adenomas [6]. In our second case, the initial histological interpretation was benign hyperplasia, and the correct diagnosis was established only after a second resection and specialist review. This pattern has been reported elsewhere in the literature [7].

Despite its rarity, parathyroid carcinoma should remain on the differential in any patient with primary hyperparathyroidism, particularly those with markedly elevated calcium or PTH, recurrent disease after apparently successful parathyroidectomy, or local symptoms such as hoarseness. The importance of this is made clear by the previously mentioned risk of metastases and a five-year survival rate of 85.5% [8]. Early involvement of the thyroid MDT and referral to a tertiary centre for specialist histopathology and, where indicated, re-exploration are important in securing the correct diagnosis and optimal outcome.

## Conclusions

Parathyroid carcinoma is a rare but clinically important cause of primary hyperparathyroidism whose clinical and biochemical presentation closely mirrors that of parathyroid adenoma. Diagnosis typically depends on histological assessment of the resected specimen. A high index of clinical suspicion, particularly in the setting of markedly elevated calcium or PTH, recurrent disease, or local symptoms such as voice change, together with early multidisciplinary input and specialist histopathological review, supports timely surgical management and the favourable outcomes seen in the two cases presented.
